# Determiners of cell fates: the tricarboxylic acid cycle versus the citrate-malate shuttle

**DOI:** 10.1093/procel/pwac026

**Published:** 2022-07-15

**Authors:** Dong Guo, Haiyan He, Ying Meng, Shudi Luo, Zhimin Lu

**Affiliations:** Zhejiang Provincial Key Laboratory of Pancreatic Disease, The First Affiliated Hospital, and Institute of Translational Medicine, Zhejiang University School of Medicine, Zhejiang University, Hangzhou 310029, China; Cancer Center, Zhejiang University, Hangzhou 310029, China; Zhejiang Provincial Key Laboratory of Pancreatic Disease, The First Affiliated Hospital, and Institute of Translational Medicine, Zhejiang University School of Medicine, Zhejiang University, Hangzhou 310029, China; Cancer Center, Zhejiang University, Hangzhou 310029, China; Zhejiang Provincial Key Laboratory of Pancreatic Disease, The First Affiliated Hospital, and Institute of Translational Medicine, Zhejiang University School of Medicine, Zhejiang University, Hangzhou 310029, China; Cancer Center, Zhejiang University, Hangzhou 310029, China; Zhejiang Provincial Key Laboratory of Pancreatic Disease, The First Affiliated Hospital, and Institute of Translational Medicine, Zhejiang University School of Medicine, Zhejiang University, Hangzhou 310029, China; Cancer Center, Zhejiang University, Hangzhou 310029, China; Zhejiang Provincial Key Laboratory of Pancreatic Disease, The First Affiliated Hospital, and Institute of Translational Medicine, Zhejiang University School of Medicine, Zhejiang University, Hangzhou 310029, China; Cancer Center, Zhejiang University, Hangzhou 310029, China

The tricarboxylic acid (TCA) cycle identified in 1937 by Hans Adolf Krebs and William Arthur Johnson (also known as the Krebs cycle or citric acid cycle) is a central route for oxidative metabolism to generate cellular energy and intermediates for the biosynthesis of amino acids, lipids, and nucleic acids (Krebs and Johnson 1937). The TCA cycle connects carbohydrate, fat, and protein metabolism and converts these nutrient catabolism-derived acetyl-CoA into carbon dioxide and water, with the production of reduced NAD^+^ (NADH), reduced flavin adenine dinucleotide (FADH2), and guanosine triphosphate (GTP). The generated NADH and FADH2 are, in turn, used by the oxidative phosphorylation pathway to generate energy-rich adenosine triphosphate (ATP) (Chakrabarty and Chandel 2021).

**Figure 1.**
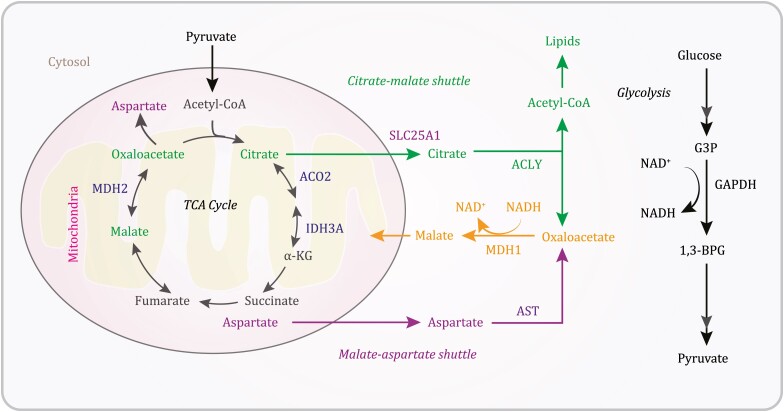
A schematic depicting the TCA cycle and anaplerotic pathways. Glycolysis-derived pyruvate is converted by the PDHC into acetyl-CoA, which joins the TCA cycle. The mitochondrial citrate-malate shuttle and the malate-aspartate shuttle couple with glycolysis to generate cytosolic malate that enters the TCA cycle. 1,3-BPG, 1,3-bisphosphoglycerate; ACLY, ATP citrate lyase; AST, aspartate transferase; G3P, glyceraldehyde 3-phosphate; IDH, isocitrate dehydrogenase; α-KG, α-ketoglutarate; MDH, malate dehydrogenase.

In addition to the generation of acetyl-CoA for the TCA cycle, catabolic pathways converge by adding intermediates to the cycle, a process known as anaplerosis. In glutaminolysis, glutamine is converted into the TCA cycle metabolite α-ketoglutarate through the activity of multiple enzymes (Zhang et al. 2017). Anaplerosis can also occur through the malate-aspartate shuttle, which translocates glycolysis-produced electrons across the semipermeable inner membrane of the mitochondria for oxidative phosphorylation in eukaryotes (Ferre and Foufelle 2007; Borst 2020). Cytosolic malate dehydrogenase 1 (MDH1) catalyzes the reaction of oxaloacetate and glycolysis-generated NADH to produce malate and NAD^+^. The malate-α-ketoglutarate antiporter imports cytosolic malate into the mitochondrial matrix and simultaneously exports α-ketoglutarate from the matrix into the cytosol. The malate is then converted by mitochondrial MDH2 into oxaloacetate, during which NAD^+^ is reduced to NADH. Oxaloacetate is subsequently catalyzed by mitochondrial aspartate aminotransferase (AST or glutamic oxaloacetic transaminase [GOT]) into aspartate, which is exported to the cytosol by the glutamate-aspartate antiporter with simultaneous importation of glutamate from the cytosol into the matrix. In the cytosol, aspartate is converted by cytosolic AST to oxaloacetate (Borst 2020). Consequently, through this shuttle, the NAD^+^ in the cytosol can be reduced again by another round of glycolysis, and the NADH in the mitochondrial matrix can be used for ATP production (Fig. 1).

Anaplerosis is also maintained by the mitochondrial citrate-malate shuttle (Fig. 1). Solute carrier family 25 member 1 (SLC25A1) translocates mitochondrial citrate to the cytosol, where ATP citrate lyase (ACLY) converts citrate into oxaloacetate as well as acetyl-CoA for *de novo* lipid biosynthesis and protein acetylation. Oxaloacetate is then converted into malate and reimported into mitochondria to support anaplerosis (Hatzivassiliou et al. 2005; Borst 2020). Through [U-^13^C] glucose-tracing metabolomics analyses, the mitochondrial citrate-malate shuttle was identified as a vital link between increased glycolysis and enhanced lipid biosynthesis in Ras-oncogene-induced hepatic tumorigenesis. Hepatocellular carcinoma cells use the citrate-malate shuttle to eliminate excessive pyruvate-dependent lactate and proton production and subsequent cell toxicity by mitochondrial pyruvate metabolism and promote cytosolic citrate-dependent lipid biogenesis and cytosolic malate-dependent anaplerosis (Lei et al. 2020). In addition, suppression of the citrate-malate shuttle inhibited cytokine-induced natural killer (NK) cell growth and proliferation and the subsequent cytotoxic effect and antitumor responses of NK cells, in which ACLY-elevated oxidative phosphorylation in mitochondria and glycolysis are crucial (Assmann et al. 2017). Thus, the mitochondrial citrate-malate shuttle is critical for some oncogenic protein- and cytokine-induced metabolic reconfigurations to sustain tumor cell growth and immune cell function, respectively.

A recent report from Arnold et al. demonstrated that the mitochondrial citrate-malate shuttle (referred to as the noncanonical TCA cycle in the report) is engaged in cell fate transitions (Arnold et al. 2022). Analyses of gene essentiality scores generated from genome-wide CRISPR screens by the DepMap project showed that TCA cycle-associated genes were separated into two distinct clusters belonging to two segments, connecting citrate production and utilization mediated by the TCA cycle and the citrate-malate shuttle, respectively (Fig. 1). In line with a previous report revealing TCA cycle-independent metabolism of glucose-derived citrate in [U-^13^C] glucose-labeled 82 non-small cell lung cancer lines (Chen et al. 2019), treatment of both human SCLC and mouse embryonic stem (ES) cells with an ACLY inhibitor or knockout of ACLY expression increased mitochondrial malate production, whereas knockout of mitochondrial aconitase (ACO2) reduced mitochondrial malate production (Arnold et al. 2022). These results suggest that citrate metabolism by ACLY provides an important alternative replenishment to the TCA cycle.

Cytosolic MDH1 catalyzes glycolysis-generated NADH and oxaloacetate to produce malate (Borst 2020). [4-^2^*H*]Glucose-tracing experiments showed that deficiency of ACLY expression in mouse ES cells increased the cytosolic NADH/NAD^+^ ratio, lowered the incorporation of hydride into malate and subsequent citrate from glycolysis-generated NADH, and decreased mitochondrial oxygen consumption. Similar results were also obtained by knocking out SLC25A1 and MDH1 (Arnold et al. 2022). These results suggest that cytosolic malate is recycled back into the mitochondria for citrate regeneration dependent on the mitochondrial citrate-malate shuttle.

Intriguingly, stem cells versus differentiated cells exhibited distinct reprogramming of the citrate-malate shuttle. Compared with myoblasts, differentiated myotubes exhibited increased incorporation of glucose-derived carbons into TCA cycle intermediates, including mitochondrial malate, compared with proliferating myoblasts, suggesting that myotubes are engaged in the TCA cycle for metabolic needs. Conversely, ACLY depletion in myoblasts mimicked this metabolic feature of myotubes, whereas ACO2 depletion had little effect on the levels of TCA cycle metabolites in myoblasts (Arnold et al. 2022), suggesting that myoblasts are engaged in the citrate-malate shuttle for proliferation.

Comparison of gene expression with TCA cycle activity showed that myogenic differentiation significantly induced most TCA cycle genes, including all subunits of the pyruvate dehydrogenase complex (PDHC). Of note, many of these genes are targets of the myogenic transcription factor MYOD. PDHC activation by dichloroacetate treatment of myoblasts and ES cells increased the incorporation of glucose-derived carbons into citrate and downstream TCA cycle metabolites, revealing a role of PDHC-metabolized pyruvate in the promotion of TCA metabolism. The exit from naive pluripotency decreased the incorporation of glucose-derived carbon into the TCA cycle while increasing the incorporation of glutamine-derived carbon, and the proportion of TCA cycle-derived malate was decreased alongside a concomitant increase in citrate production from cytosolic intermediates, thus indicating a switch from the TCA cycle to the citrate-malate shuttle. ACLY depletion increased the expression of the naive pluripotency genes Nanog, Esrrb, and Rex1, impaired the induction of the differentiation marker Sox1, and prevented ES exit of pluripotency. Consistently, depletion of ACLY or SLC25A1, which did not affect the viability of naive pluripotent ES cells, reduced the aspartate pool and impaired protein synthesis and cell proliferation and viability as cells exited the naive pluripotent state (Arnold et al. 2022), suggesting a requirement of the citrate-malate shuttle for exit from naive pluripotency.

These results imply that increased glycolysis by regenerating cytosolic NAD^+^ and citrate-dependent downstream metabolism, which are promoted by the citrate-malate shuttle, play a role in cell fate determination.

Fine-tuned mitochondrial activity is critical for diversified cellular activities. The regulation of mitochondrial activity and anaplerosis can be dynamic and context dependent. Activation of receptor tyrosine kinase and expression of oncogenic K-Ras and B-Raf in glioblastoma cells suppressed mitochondrial metabolism of glycolysis-derived pyruvate through phosphoglycerate kinase 1 (PGK1)-mediated phosphorylation and inhibition of pyruvate dehydrogenase kinase 1 (PDHK1), with a concomitant increase in glutaminolysis (Li et al. 2016). Under low glucose conditions, choline kinase (CHK) α2-mediated lipolysis of lipid droplets promoted fatty acid oxidization in mitochondria for cell survival (Liu et al. 2021). While both the citrate-malate and malate-aspartate shuttles can couple glycolysis with the TCA cycle by regenerating cytosolic NAD^+^ required to sustain glycolysis and supplying mitochondrial NADH for ATP production, the citrate-malate shuttle provides additional advantages for the cytosolic acetyl-CoA production required for lipid biosynthesis and protein acetylation, which are essential for cell proliferation. The preferential or switching usage of the particular shuttle or anaplerotic pathway is precisely regulated in cells in response to intracellular or extracellular signaling or stimuli. As the cellular energy- and metabolic intermediates-manufacturing plants and signaling organelles (Chakrabarty and Chandel 2021), mitochondria, similar to Rome with all roads lead to, join the intracellular network by interchanging signaling, metabolic intermediates, and building blocks for biosynthesis and supplying ATP, which consequently dictate cell function, state, and fate.
